# Social mass gathering events influence emergency medical services call volume

**DOI:** 10.3389/fpubh.2024.1394384

**Published:** 2024-05-30

**Authors:** Calvin Lukas Kienbacher, Harald Herkner, Feven Alemu, Jason M. Rhodes, Norah Al Rasheed, Ibrahem Aldeghaither, Esam Barnawi, Kenneth Alan Williams

**Affiliations:** ^1^Department of Emergency Medicine, Division of Emergency Medical Services, Warren Alpert Medical School of Brown University, Providence, RI, United States; ^2^Department of Emergency Medicine, Medical University of Vienna, Vienna, Austria; ^3^Rhode Island Department of Health, Division of Emergency Medical Services, Providence, RI, United States; ^4^Department of Emergency Medicine, King Abdulaziz Medical City, Riyadh, Saudi Arabia; ^5^Department of Emergency Medicine, Prince Mohammed Bin Abdulaziz Hospital, Riyadh, Saudi Arabia; ^6^Pediatric Emergency Department, Emergency Medicine Administration, King Fahad Medical City, Riyadh, Saudi Arabia

**Keywords:** emergency medical services, prehospital emergency medicine, mass gathering events, public health, weather

## Abstract

**Background:**

Prior literature suggests that mass gathering events pose challenges to an emergency medical services (EMS) system. We aimed to investigate whether events influence EMS call rates.

**Materials and methods:**

This study is a retrospective review of all primary response ambulance calls in Rhode Island (US) between January 1st, 2018 and August 31st, 2022. The number of EMS calls per day was taken from the state’s EMS registry. Event data was collected using a Google (Google LLC, Mountain View, CA) search. We used separate Poisson regression models with the number of ambulance calls as the dependent and the social event categories sports, agricultural, music events, and public exhibitions as independent variables. All models controlled for the population at risk and the period of the COVID−19 pandemic. Results are presented as increases or decreases in calls per 100,000 inhabitants from the mean over the study period.

**Results:**

The mean number of daily EMS calls was 38 ± 4 per 100,000 inhabitants. EMS encountered significantly more missions on days with music events (+3, 95% CI [2; 3]) and public exhibitions (+2, 95% CI [1; 2]). In contrast, days with agricultural events were associated with fewer calls (−1, 95% CI [−1; 0]). We did not find any effect of sports events on call rates.

**Conclusion:**

Increased ambulance call volumes are observed on days with music events and public exhibitions. Days with agricultural events are associated with fewer EMS calls.

## Introduction

Mass gathering events are defined as events that could be strenuous for a community’s resources (1). They can occur in a planned (e.g., concerts, public exhibitions, political marches) or unplanned manner (e.g., natural disasters). The former is usually influenceable well in advance and serve a social or recreational purpose. On the other hand, prior literature suggests that such social mass gathering events may compromise emergency medical services (EMS) capabilities, which makes them particularly interesting for research (2–4). One possible reason behind this phenomenon might be that an increased call volume leads to fewer ambulances being available in the right spot at the right time (2). Another reason might be physical barriers, such as roadblocks, which lead to detours and diversions (2). From an operational perspective, the event may pose a risk for its participants, individuals not involved but in the same catchment area, and the responsible EMS system (5–7). Although most individuals present with minor complaints, nearby emergency departments may be confronted with unusually high numbers of patients within short periods of time (8–10). This issue can become even more important with the increasing duration of an event (8–10). Overcrowding at the venue may make participants feel uncomfortable, which can lead to panic and physical aggression (9). Medical problems may be triggered by sleep, food, or fluid deprivation (9).

Aside from social events, public behavior might also change substantially during holidays and weekends, i.e., days when the majority of a population is off from work. Anticipating the effect of social events and days off from work on ambulance call rates well in advance might help to allocate resources properly (9).

Rhode Island is a well-suited model region for EMS research. The smallest state in the United States (US), with one million inhabitants, has an urban center surrounded by suburban areas (11). It is one of New England’s most vibrant cultural hotspots, with recreational and public events of various kinds taking place regularly. The sociodemographic characteristics of Rhode Island’s population are around the national US average (11). The state’s EMS system is mainly municipal and fire department based.

Prior data showed that prehospital systems greatly differ globally (12). This includes the number of ambulances available for a given number of citizens, the capabilities of the units dispatched (e.g., basic or advanced life support ambulances), and the clinicians’ expertise. While paramedics treat critical patients autonomously in many Anglo-Saxon countries, central Europe relies on EMS physicians for those who are severely ill (12). These factors must be considered when conceptualizing medical care for an event.

Planning events thoroughly often necessitates coverage with on-site healthcare personnel. The *Purple Guide* is an internationally recognized guideline for organizers (13). It recommends medical risk assessment by looking at the nature of the happening (13). The individual risk classes are related to the physical activities performed at the event as well as other factors such as the potential consumption of alcoholic beverages and psychotropic substances (9, 13). An important cornerstone of keeping the population safe is the avoidance of the overuse of EMS resources serving a community (1, 14–17). The implications are manifold and range from routine emergency medical care and infectious disease control at the event venue to the communication between participants, organizers, and healthcare officials (14–16). In this context, it is yet unclear whether the nature of an event can be used as a surrogate for the strain of an entire EMS system on the day the happening occurs.

Whether people participate in an event may be influenced by the weather, which includes precipitation and temperature (6, 9). Prior investigations have demonstrated that perceived temperatures, i.e., how temperatures are felt by humans, reflect physical strain better than their crude values (18, 19). Established formulae can be used to calculate the perceived temperature from crude temperature, humidity, and wind speed (20, 21). National Oceanic and Atmospheric Administration (NOAA) and Iowa State University provide the daily averages of these parameters (22, 23). The US Census Bureau, on the other hand, publishes yearly statistics on population size (24).

While some characteristics may differ between various EMS systems, Rhode Island shares many common risk factors with other settings. These include that there are days off from work for a large part of the population, various types of events taking place, and climate (6, 13).

We aimed to investigate the strain of different natures of social events and days off from work on an EMS system. Furthermore, we explore the influence of weather on ambulance call volume during the respective days.

## Materials and methods

We retrospectively reviewed the daily number of medical 9–1-1 calls between January 1st, 2018, and August 31st, 2022, the most recent date for which data was available. The number of EMS calls per day was acquired from the statewide EMS registry. The dates and natures of the social mass gathering events were acquired via a thorough Google (Google LLC, Mountain View, CA) search performed by four researchers. A comprehensive list of all social mass gathering events and their respective dates are provided in Supplement 1. To facilitate analysis, we pooled the nature of the happenings into four categories: sports events, agricultural events, music events, and public exhibitions. Public holidays and weekends (Saturdays and Sundays) were considered “days off from work.”

We developed Poisson regression models with the number of EMS calls per day as the dependent variable. The categories of events as well as days off from work were alternately used as independent variables. The Poisson regression model allowed us to control for the population at risk (inhabitants of Rhode Island, as provided by the US Census Bureau annually). We present unadjusted estimates of the respective effects.

In a secondary analysis, we investigated the association between EMS call volume, social risk factors, and weather again using Poisson regression. The daily average apparent temperature was calculated from daily average parameters provided by the NOAA and Iowa State University using the Magnus formula and Steadman equation for outside areas in the shade (20, 21). We then defined the first and fifth quintiles of the apparent temperature over the study period as low and high and the values in-between as the baseline. The regression models again controlled for the population at risk and for the period of the COVID-19 pandemic as a covariate.

A day served as the unit of analysis. All results are presented as calls per 100,000 inhabitants. Regarding changes in EMS call volume, positive numbers indicate increases whereas negative numbers indicate decreases in calls per 100,000 inhabitants in relation to the mean call rate over the study period.

A two-sided *p*-value of <0.05 was considered statistically significant. We used MS Excel version 16.77 (Microsoft Corp., Redmond, WA) and StataSE 18.0 (StataCorp, College Station, TX) for data curation and analysis, respectively. The local ethics committee approved the study protocol with an exemption from full review (vote #2022–17). The project follows the principles of the Declaration of Helsinki. The RECORD (25) statement for this manuscript can be found in Supplement 2.

## Results

During our study period of 1,704 days, there were 695,535 EMS calls. The mean number of calls per day was 38 ± 4 per 100,000 inhabitants. Social mass gathering events took place on 286 days (17% of the observation period’s days). Their characteristics can be found in Table 1.

**Table 1:** Calls per 100,000 inhabitants on days with social risk factors over the study period (1,704 days).

Risk factor, *n* (%)	Call rate, mean (SD)
Sports Events, 165 (10)	38 (3)
Agricultural Events, 50 (3)	37 (5)
Music Events, 55 (3)	41 (3)
Public Exhibitions, 28 (2)	40 (4)
Weekend days, 486 (29)	36 (4)
Days off from work, 550 (32)	36 (4)

n, number of days with risk factor; SD, standard deviation.

EMS encountered significantly more missions on days with music events (+3 calls per 100,000 inhabitants, 95% CI [2; 3]) and public exhibitions (+2, 95% CI [1; 2]). Days with agricultural events were significantly associated with decreased call volumes (−1, 95% CI [−1; 0]). A similar effect applied to days off from work and weekends (−2, 95% CI [−2; −2]). Table 2 presents the findings in detail.

**Table 2:** Social risk factors.

Risk factor	Change in call rate [95% CI]
Sports Events	0 [0; 0]
Agricultural Events	−1 [−1; 0]*
Music Events	3 [2; 3]**
Public Exhibitions	2 [1; 2]**
Weekend days	−2 [−2; −2]**
Days off from work	−2 [−2; −2]**

Values are based on calls per 100,000 inhabitants, where positive and negative numbers indicate increases and decreases, respectively. *p < 0.01, **p < 0.001. n, number of days with risk factor over study period, 95% CI, 95% confidence interval.

Our regression models showed significant reductions in ambulance call volume on days with low apparent temperatures and agricultural events (−9, 95% CI [−12; −5]) but an increase on days with sports events (+1, 95% CI [0; 2]). High apparent temperatures in combination with music events (+2, 95% CI [1; 2]) as well as public exhibitions (+2, 95% CI [1; 3]) were associated with higher call rates. Snow, rain, and fog were associated with fewer calls on days with agricultural events (snow: -2, 95% CI [−3; 0]; rain: -1, 95% CI [−2; −1]; fog: -2, 95% CI [−3; −1]), but with more calls on days with music events (snow: +6, 95% CI [3; 9]; rain: +4, 95% CI [3; 5]; fog: +4, 95% CI [3; 5]). Weekends and days off from work were generally associated with fewer calls. Furthermore, EMS encountered more calls on rainy days with sports events (see Table 3).

**Table 3:** Social risk factors and weather.

Risk factor	Change in call rate [95% CI]					
	Low AT, *n* = 340 (20%)	High AT, *n* = 341 (20%)	Snow, *n* = 273 (16%)	Rain, *n* = 686 (40%)	Fog, *n* = 729 (43%)	Thunderstorm, *n* = 109 (6%)
Baseline	−1 [−1; -1]***	3 [3; 3]***	-1 [−2; -1]***	0 [−1; 0]***	0 [0; 0]	2 [1; 2]***
Sports events	1 [0; 2]**	1 [0; 1]**	0 [−1; 1]	0 [0; 1]*	0 [0; 0]	1 [0; 3]
Agricultural events	−9 [−12; −5]***	0 [−1; 0]	−2 [−3; 0]*	−1 [−2; −1]***	−2 [−3; −1]***	−1 [−3; 0]
Music events	0 [−4; 4]	2 [1; 2]***	6 [3; 9]***	4 [3; 5]***	4 [3; 5]***	2 [0; 4]
Public Exhibitions	−1 [−3; 1]	2 [1; 3]***	0 [−2; 2]	1 [−1; 2]	0 [−1; 1]	n/a
Weekends	−3 [−3; −2]***	−2 [−2; −2]***	−2 [−3; −2]***	−2 [−2; −2]***	−2 [−3; −2]***	−2 [−3; −1]***
Days off from work	−3 [−3; −2]***	−2 [−2; −2]***	−2 [−3; −2]***	−2 [−3; −2]***	−2 [−3; −2]***	−2 [−3; −1]***

Values are based on calls per 100,000 inhabitants, where positive and negative numbers indicate increases and decreases, respectively. *p < 0.01, **p < 0.001. AT, apparent temperature; n, number of days with risk factor; 95% CI, 95% confidence interval.

We did not find any significant associations between EMS call volume and sports events on days with snow, fog, or thunderstorms, and agricultural events during high apparent temperatures or thunderstorms. Furthermore, our findings suggest no associations between ambulance call rate and music events on days with low apparent temperatures and thunderstorms or public exhibitions a day with low apparent temperatures, snow, rain, or fog.

## Discussion

Our data from a state-wide survey suggest that music events and public exhibitions are associated with an increased EMS call volume. Fewer calls were encountered on weekends, days off from work, and days with agricultural events.

One might argue that the changes in EMS call volume on event days are rather small. However, we found a 7% increase in the statewide call rate on days with music events, which took place 55 times over the study period. This corresponds to approximately 1,500 ambulance missions attributed to the music events throughout our observation period. Assuming that 2 EMS clinicians would be able to respond to 10 calls in a 12-h shift, this translates to 300 shifts for one qualified person. Considering the current median wage of a prehospital clinician in the US of US $ 18/h (26), this corresponds to roughly US $ 65,000 in EMS expenses, exclusively for salaries. A similar effect applies to public exhibitions. In addition to these economic aspects, the increase in call volume may impair timely care for the entire population of a region (17). Additionally, knowledge of the impact events have on EMS call volume may inform EMS agencies contracted to provide coverage at or near these events, allowing them to estimate call volume and allocate assets efficiently.

Data on the impact of events on EMS call volume are sparse. However, prior research indicates that mass gatherings might lead to a delay in patient care in both hospital and pre-hospital settings (17). This circumstance can be partially explained by the increased capacity utilization of ambulance resources. However, this effect is likely dependent on the first aid capacities at an event (9, 10). Clinicians at a venue might be able to treat patients with minor complaints, thereby avoiding hospitalization (9, 10). In turn, individuals who are hospitalized despite on-site treatment might be sicker than patients encountered at events without any coverage from medical professionals (17).

A study from San Francisco suggests that a community’s baseline ambulance call rate *per capita* is higher than that at an event venue (27). However, the respective findings are derived from county and census tract data, not from a statewide registry. This might be relevant, as mutual aid, i.e., EMS resources helping out in other communities than they primarily serve, is common in many areas (9, 28). Regarding medical emergencies, researchers from Japan also found no association between a major sports event and out-of-hospital cardiac arrest (29).

Important factors influencing the call rate are the geographic distance between an event venue and adequate healthcare institutions, as well as the number of available EMS units (9). Long transport times in resource-sparse settings generate a misleadingly low call rate, as ambulance units can generally only respond to one case at a time (1). Vice versa, short transport intervals and abundant resources can favor high call rates (1). Rhode Island’s EMS network is rather close-meshed and ground-bound travel distances are fairly short.

## Limitations

Our study has several limitations. First, we included all EMS calls within the state during a given day in our analysis and did not differentiate between geographical areas. However, Rhode Island is a small state and comparable to many major cities worldwide. Second, we did not consider the audience profile of the events. Third, we did not include logistic factors in our calculations, such as whether the event took place indoors or outdoors, and the medical personnel on-site. However, in our setting, EMS resources at an event are usually limited to a few units that rarely transport patients. Hospitalization is often initiated via 911.

Another important point is the generalizability of our findings. EMS systems and the capabilities of prehospital resources differ around the world, which might also influence the strain an event poses for ambulance resources (12). In physician-based systems, a single critical case may result in at least two vehicles responding, as doctors commonly work in liaison systems using independent response cars, which cannot transport patients (12). The consequence is that the number of ambulance missions increases with the acuity of patients, which was not the case in this study (12).

## Conclusion

Increased ambulance call volumes are observed on days with music events and public exhibitions. Weekends, days off from work, and days with agricultural events are associated with fewer EMS calls. Weather influences the effect of events on ambulance call rate.

## Data availability statement

The raw data on events supporting the conclusions of this article will be made available by the authors, without undue reservation. Data on daily EMS call volume cannot be shared due to IRB restrictions.

## Ethics statement

The studies involving humans were approved by Rhode Island Department of Health. The studies were conducted in accordance with the local legislation and institutional requirements. The ethics committee/institutional review board waived the requirement of written informed consent for participation from the participants or the participants’ legal guardians/next of kin because low-risk retrospective data survey.

## Author contributions

CK: Writing – original draft, Visualization, Validation, Supervision, Software, Resources, Project administration, Methodology, Investigation, Formal analysis, Data curation, Conceptualization. HH: Writing – review & editing, Validation, Supervision, Software, Resources, Methodology, Investigation, Formal analysis, Conceptualization. FA: Writing – review & editing, Validation, Resources, Methodology, Investigation. JR: Writing – review & editing, Validation, Supervision, Software, Resources, Project administration, Methodology, Investigation, Data curation, Conceptualization. NR: Writing – review & editing, Validation, Resources, Investigation, Data curation. IA: Writing – review & editing, Validation, Software, Resources, Investigation, Data curation. EB: Writing – review & editing, Validation, Resources, Investigation, Data curation. KW: Writing – review & editing, Validation, Supervision, Resources, Project administration, Methodology, Investigation, Conceptualization.
